# Identification of a Novel Clinical Phenotype of Severe Malaria using a Network-Based Clustering Approach

**DOI:** 10.1038/s41598-018-31320-w

**Published:** 2018-08-27

**Authors:** Ornella Cominetti, David Smith, Fred Hoffman, Muminatou Jallow, Marie L. Thézénas, Honglei Huang, Dominic Kwiatkowski, Philip K. Maini, Climent Casals-Pascual

**Affiliations:** 1 Wolfson Centre for Mathematical Biology, Mathematical Institute, University of Oxford, Oxford, UK; 2 London School of Hygiene and Tropical Medicine, Keppel Street, London, UK; 3 Department of Computer Science, University of Oxford, Oxford, UK; 4 MRC Unit, The Gambia, Serekunda, Gambia; 5 Wellcome Trust Centre for Human Genetics, University of Oxford, Oxford, UK; 6 ISGlobal, Hospital Clínic i Provincial de Barcelona, Centre Diagnòstic Biomèdic- Universitat de Barcelona, Barcelona, Spain; 7 Present Address: Nestlé Institute of Health Sciences, Lausanne, Switzerland; 8 Present Address: XL Catlin, London, UK

## Abstract

The parasite *Plasmodium falciparum* is the main cause of severe malaria (SM). Despite treatment with antimalarial drugs, more than 400,000 deaths are reported every year, mainly in African children. The diversity of clinical presentations associated with SM highlights important differences in disease pathogenesis that often require specific therapeutic options. The clinical heterogeneity of SM is largely unresolved. Here we report a network-based analysis of clinical phenotypes associated with SM in 2,915 Gambian children admitted to hospital with *Plasmodium falciparum* malaria. We used a network-based clustering method which revealed a strong correlation between disease heterogeneity and mortality. The analysis identified four distinct clusters of SM and respiratory distress that departed from the WHO definition. Patients in these clusters characteristically presented with liver enlargement and high concentrations of brain natriuretic peptide (BNP), giving support to the potential role of circulatory overload and/or right-sided heart failure as a mechanism of disease. The role of heart failure is controversial in SM and our work suggests that standard clinical management may not be appropriate. We find that our clustering can be a powerful data exploration tool to identify novel disease phenotypes and therapeutic options to reduce malaria-associated mortality.

## Introduction

Severe malaria (SM) is a major public health problem and a complex disease. Worldwide, 3.3 billion people live in areas where malaria is transmitted by infected anopheline mosquitoes. Despite recent improvements in the implementation of effective control measures in some countries, in 2016 the estimated number of clinical malaria cases globally was 216 million, with 445,000 deaths (1).

The definition of severe malaria proposed by the World Health Organization (WHO) was designed to capture the majority of children at risk of dying and thus it prioritizes sensitivity over specificity. In sub-Saharan Africa, children with coma (cerebral malaria) and/or respiratory distress are at the highest risk of death. These clinical syndromes capture a heterogeneous population that possibly reflect diverse pathophysiological processes. Critically, the current WHO classification of SM fails to capture this heterogeneity and thus treatment allocation based on this definition may have undesired consequences. Most adjuvant treatments proposed to date have consistently failed to improve patient outcome.

A systems approach to medicine applies mathematical and computational models of biological systems to make predictions about complex biological functions^1^. For example, high-dimensional data from clinical studies or data generated with high-throughput technologies can be represented by networks. The structure of these networks can be studied to help develop intuition about how clinical presentations are related and to how network structure correlates with biological function or clinical phenotype^2,3^.

**Figure 1:**
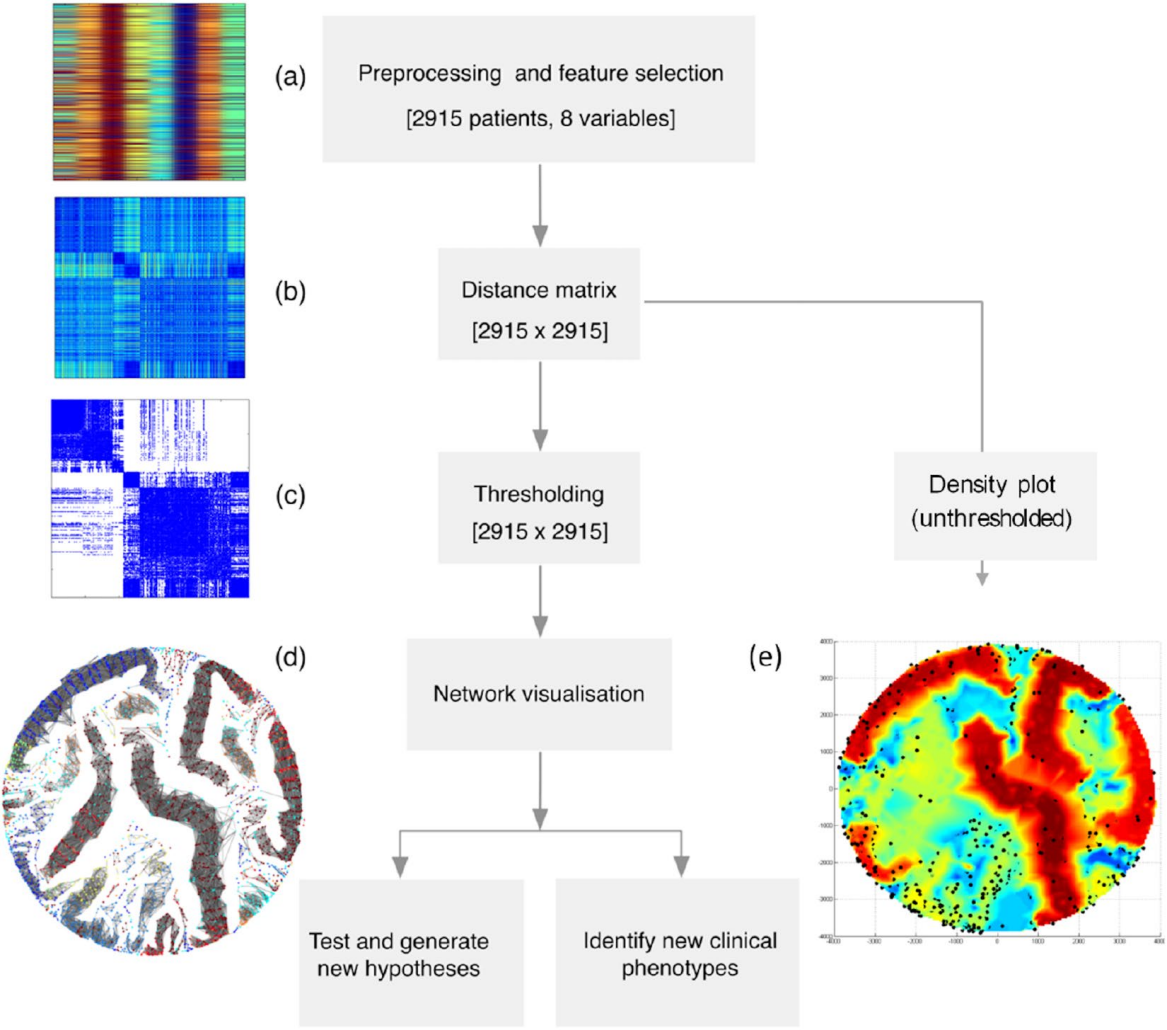
Study workflow. (**a**) *Pre-processing of data and feature selection*: To allow data on different scales to be compared and derive meaningful distances between patients, clinical variables were normalized prior to analysis. To minimize the noise introduced by redundant variables, the most informative clinical features were selected based on their ability to account for variation in the data using sparse PCA (inverse power method^36^). (**b**) Definition of the distance matrix: The distance matrix contains all the pairwise Euclidean distances between any pair of points (patients) in the dataset. Distances between data points corresponding to different patients were derived based on the reduced set of variables selected in a). (**c**) Clustering Coefficient-based thresholding: In order to find those clusters that maximize similarities within clusters and differences between clusters, a distance threshold was derived as a fraction of the maximum pairwise distance. This information was used to determine which pairs of nodes (patients) were linked in the network (see methods) (**d**). Proximity of patients in the feature spaced was based on unthresholded inter-patient distances and represented as a density heatmap (**e**).

We hypothesized that a rational unbiased approach to classify disease, which takes into account clinical heterogeneity, may improve our understanding of disease pathogenesis and identify novel therapeutic targets. We have investigated the use of a network-based approach to identify biologically meaningful phenotypes that depart from the current clinical definition in 2,915 Gambian children admitted to hospital with SM (Fig. 1).

## Results

### Clinical Features-Selection and Generation of a Network of 2,915 Children with Severe Malaria

A well curated clinical dataset of 2,915 Gambian children with SM was used to identify a reduced set of clinical features to derive biologically meaningful distances between patients (Table 1 and sTable 1)^4,5^. We used sparse principal component analysis (sPCA) to select the clinical features that best separated different patient groups (clusters) without prior knowledge of the underlying clinical syndrome^6^. We observed that approximately 60% of the variability of the data was explained by just 13 variables of a total of 46 clinically relevant variables included in the analysis (sFig. 1). The subsequent addition of clinical features selected by sPCA had a marginal impact on the percentage of variability explained. To derive a network of patients in a biologically meaningful space, we then selected only those variables that were significantly associated with an unambiguous clinical outcome (death) based on statistical significance and low collinearity (sFig. 1b). Only eight variables significantly associated with mortality were finally selected to derive distance measures between patients (sTable 2). These variables broadly captured three of the most relevant pathogenic mechanisms of SM, namely impairment of brain function (Blantyre coma score^7^, seizures during admission, tonic seizures and unusual sleepiness), impairment of respiratory function (deep breathing, use of accessory muscles during respiration and intercostal recession) and anemia (measured by hemoglobin concentration).

**Table 1:** Baseline characteristics of the population studied.

		Observations	Value
Age in months, median (IQR)	Median (IQR)	2,695	44 (27–71)
Sex (female)	%	2,695	47.9
Temperature (°C)	Mean (SD)	2,674	38.1 (1.01)
Hemoglobin (g/dL)	Mean (SD)	2,695	6.57 (2.48)
Parasite density (parasites/µL)	Geometric mean (95%CI)	2,695	33,049 (30,692–35,588)
Coma score	%	[2,695]	
0		41	2.63
1		261	9.68
2		694	25.7
3		520	19.2
4		401	14.8
5		748	27.7
Respiratory distress	%	2,695	40.8
Severe anemia	%	2,695	23.8
Hypoglycemia	%	2,042	21.9
Hepatomegaly	%	2,662	38.8
Splenomegaly	%	2,662	16.8
Transfusion	%	2,695	48.8

Clinical variables were defined as follows: Severe anemia (with any parasite density), Hb < 5 g/dL or PCV < 15; Respiratory distress, abnormal respiratory pattern (respiratory pattern values > or = 3), grunting or use of accessory muscles of respiration, or abnormally deep (acidotic) breathing; Hypoglycemia ≤ 2.2 mM; Hepatomegaly > 2 cm below right costal margin; Splenomegaly > 2 cm below left costal margin.

**Figure 2:**
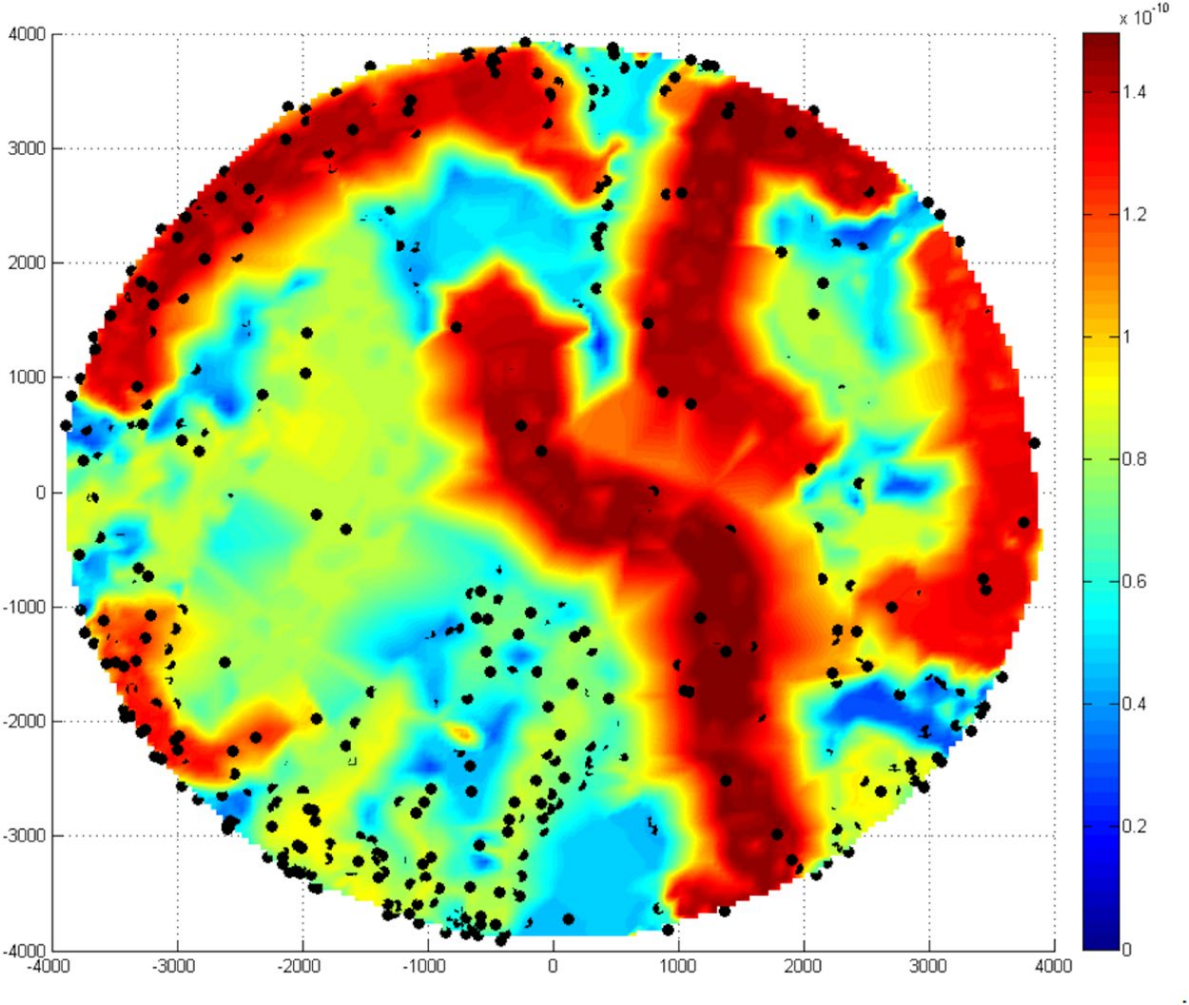
Clinical heterogeneity of severe malaria. Density heatmap shows the distribution of patients in the 8-dimensional feature space. Higher density values (in red) indicate closer proximity of patients in this feature space. Black dots indicate SM patients who died.

**Figure 3:**
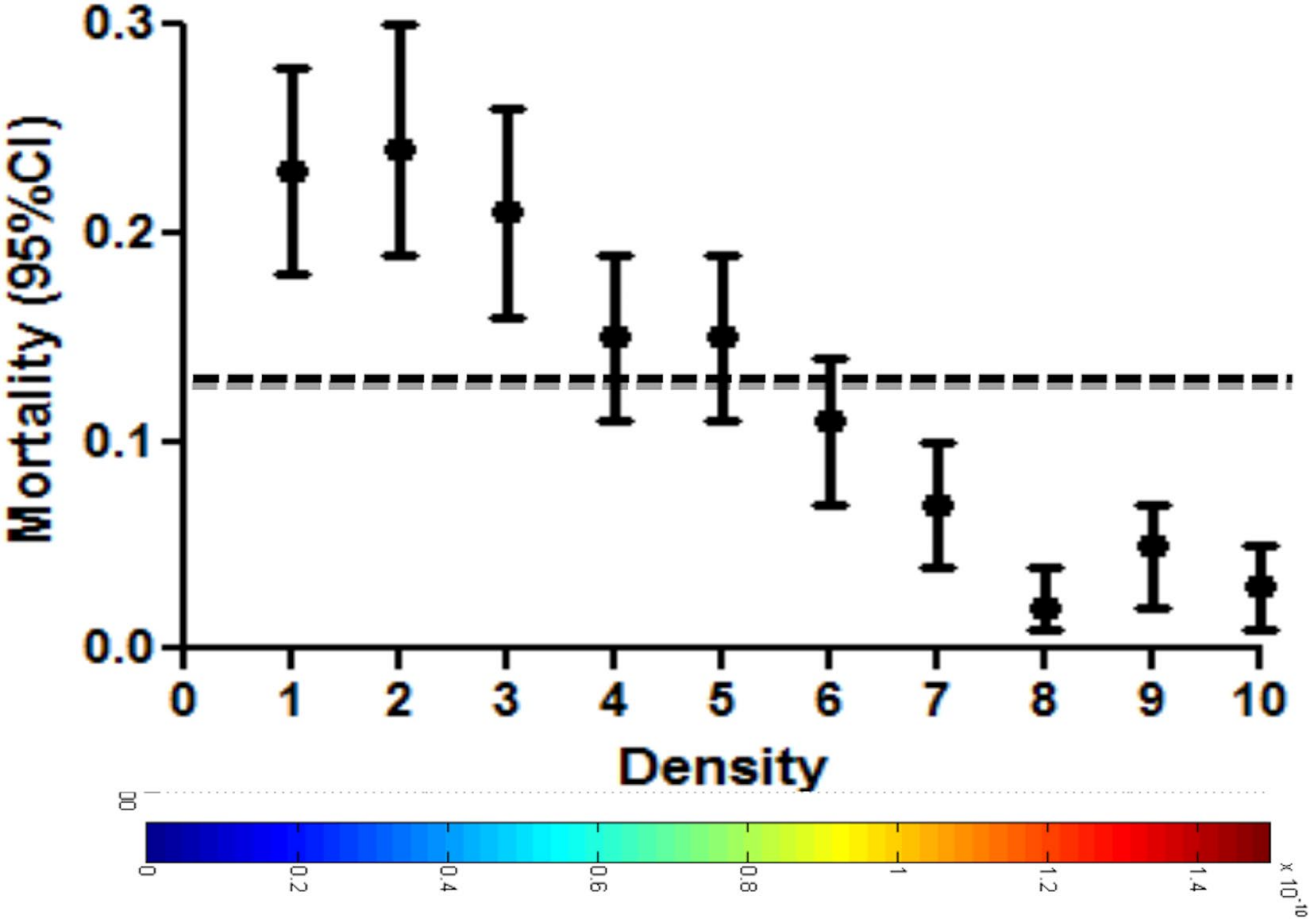
Clinical heterogeneity and mortality in severe malaria. Quantile distribution of patient density (10 quantiles) and mortality rates and 95% confidence intervals in children with SM. Dotted line indicates average mortality in the population studied.

We then used a Gaussian kernel function to assign a density to each patient depending on the distance to all other patients irrespective of the patient’s original cluster-assignment (Fig. 2). The patient distribution was plotted in a density heat map where areas of high density indicated clinical phenotypes with a composition of patients with highly similar clinical features and areas with low density represented more heterogeneous phenotypes. The density estimation showed that patients in low-density areas were highly correlated with patients with multiple SM syndromes (P < 0.001). The lowest density corresponded to patients with all three SM syndromes, whereas the SM syndrome with the highest density (homogeneity) was severe malarial anemia (sFig. 6). We observed that mortality was significantly higher in those phenotypes with lower Gaussian density (P < 0.001) (Fig. 3).

### Validation of Cluster Distribution of the Thresholded Network of Children with Severe Malaria

A distance threshold was used for network analysis and visualization purposes. The thresholded network included 238 clusters. Of these, only 19 clusters contained more than 20 patients, with case fatality rates that ranged from 0% to 53%. We reasoned that if this set of clusters were a random partition, the mortality of clusters would show a tendency towards the average mortality of the overall study population. To test this, we preserved the topology of the original network but randomly shuffled the patient mortalities associated with each node. In 12 of the 19 clusters identified in the original network, the proportion of deaths was significantly higher than that observed for networks with a shuffled relationship between nodes and patients (sFig. 4). Similarly, to verify that the clusters identified were not a peculiarity of our method, we checked cluster composition using a standard clustering method (hierarchical clustering)^8,9^. The comparison of the network clusters with clusters built from the same set of clinical features using hierarchical clustering method showed a high level of agreement (Rand Index = 0.98) (sFig. 5).

### Identification and Biological Validation of Clusters Identified in a Network of Patients with Respiratory Distress and Severe Anemia

**Figure 4:**
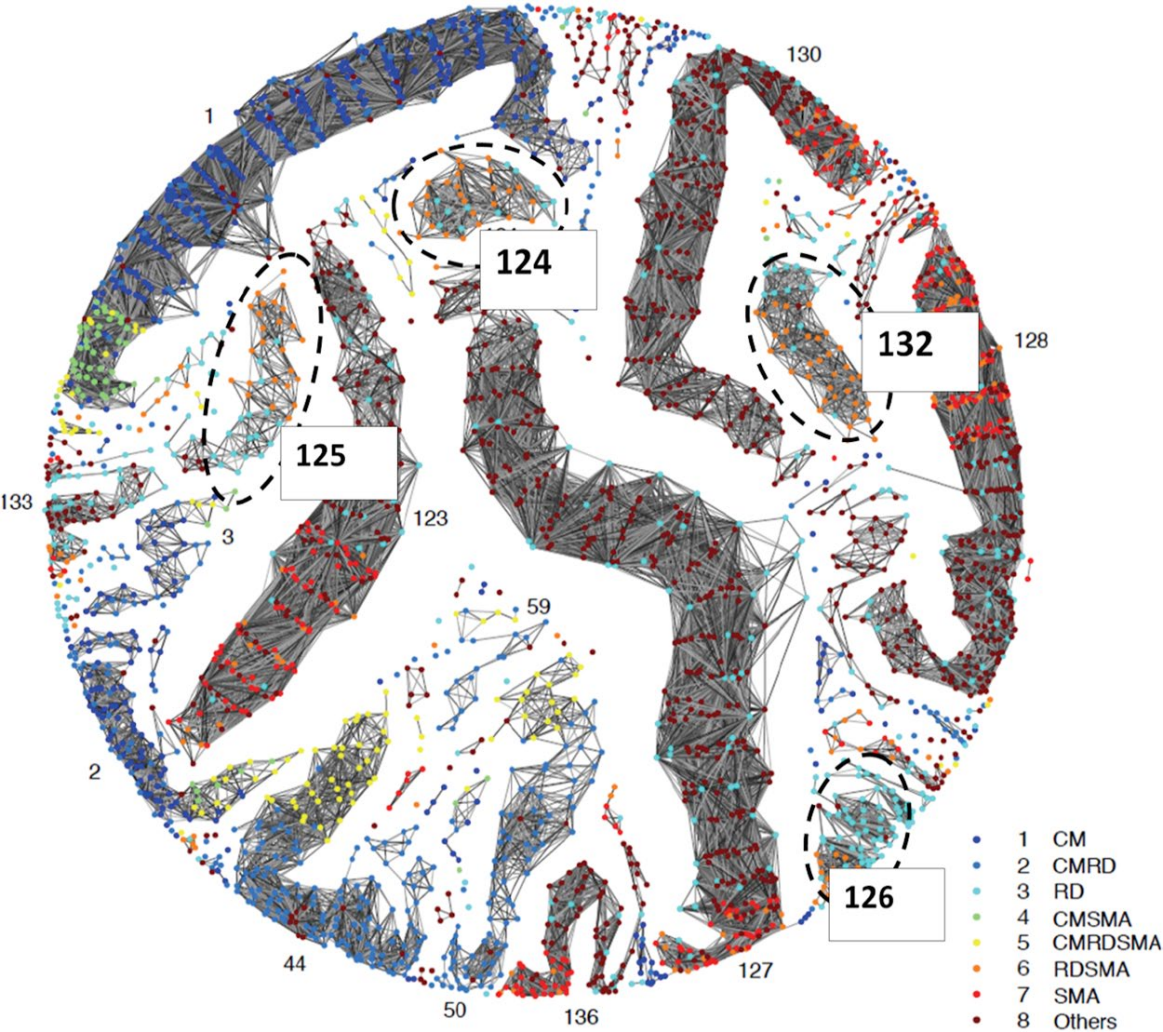
Network visualization of 2,915 children with severe malaria. A network visualization of the distinct clusters (connected components after thresholding) of patients. The nodes are coloured according to the WHO definition. The four clusters of respiratory distress and severe anemia that were identified in the network (clusters 124, 125, 126 and 132) appeared segregated despite similar WHO-defined clinical composition. CM: cerebral malaria; CMRD: cerebral malaria with respiratory distress; RD: respiratory distress; CMSMA: cerebral malaria with severe malarial anemia; CMRDSMA: cerebral malaria, respiratory distress and severe malarial anemia; RDSMA: respiratory distress with severe malarial anemia; SMA: severe malarial anemia. ‘Others’ include children who did not meet criteria to be included as severe malaria syndromes.

**Figure 5:**
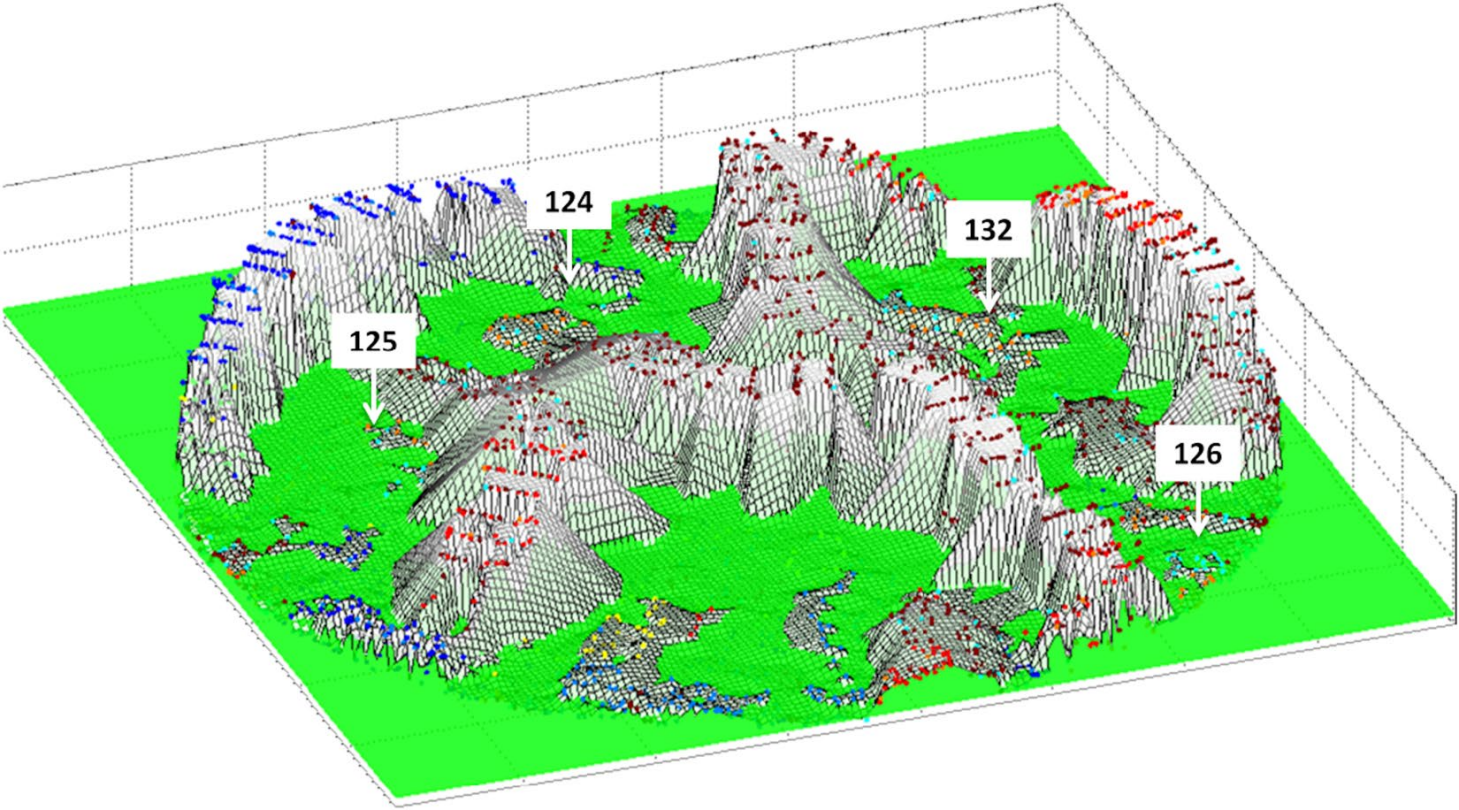
Clinical heterogeneity and cluster visualization of severe malaria. A 3D plot to visualize the relationship of cluster density showing clinical heterogeneity (lower height) and the distribution of patients in the clusters as indicated in Fig. 4. Cluster height indicates patients presenting with a similar set of clinical signs. A video to visualize and navigate this figure can be accessed online.

To compare the clusters in the thresholded network with the distribution predicted by the standard WHO definition, patients in the network were colour-coded using the WHO classification of the different SM syndromes^10^, namely cerebral malaria (CM), respiratory distress (RD), severe malarial anemia (SMA) or a combination of these syndromes (Figs 4 and 5).

The phenotypic analysis of the network revealed an evident trend for patients to cluster according to the standard definition of the SM syndromes proposed by the WHO. However, some patient clusters, despite having the same composition of standard phenotype allocations, were further separated into new groups (Fig. 4). In particular, we wondered why children with RD, or SMA with RD, were segregated into four different clusters (clusters 124, 125, 126 and 132 in Fig. 4, dashed ovals) when we might have expected them to lie in a single cluster. To provide a biological validation for this partition of patients, we used liquid chromatography tandem mass-spectrometry (LC-MS/MS) to characterize the plasma proteome of samples from patients included in these clusters. We found that the differences observed in the plasma proteome were larger across clusters than within clusters (sFig. 7). The results from the proteomic analysis support the notion that patients belonging to different clusters are biologically different but left the physiological interpretation open (details of the differentially regulated protein can be found in sFig. 7). To investigate this further we both returned to our full feature space and performed more experiments.

### Clinical Validation of Phenotypes of SM associated with Respiratory Distress and Severe Anemia

We sought to account for the clinical difference between the four clusters. The clinical features that determined the segregation of the 4 clusters of SM with RD, namely degree of consciousness (Blantyre coma score) and abnormal sleepiness, were clinically non-specific and insufficient to gain any insight into the underlying mechanism of disease (Table 1). Despite this, clusters 124 and 132 are clinically very similar for a number of indicators (Table 1) and yet have very different mortality rates. We sought to determine what other clinical features best accounted for the segregation of the 4 clusters and used sPCA including all 46 clinical variables. This analysis revealed that hepatomegaly (liver enlargement) was the clinical feature that best explained the separation of these clusters (children in cluster 132 have both a higher rate of hepatomegaly and a higher death rate than in cluster 124).

**Figure 6:**
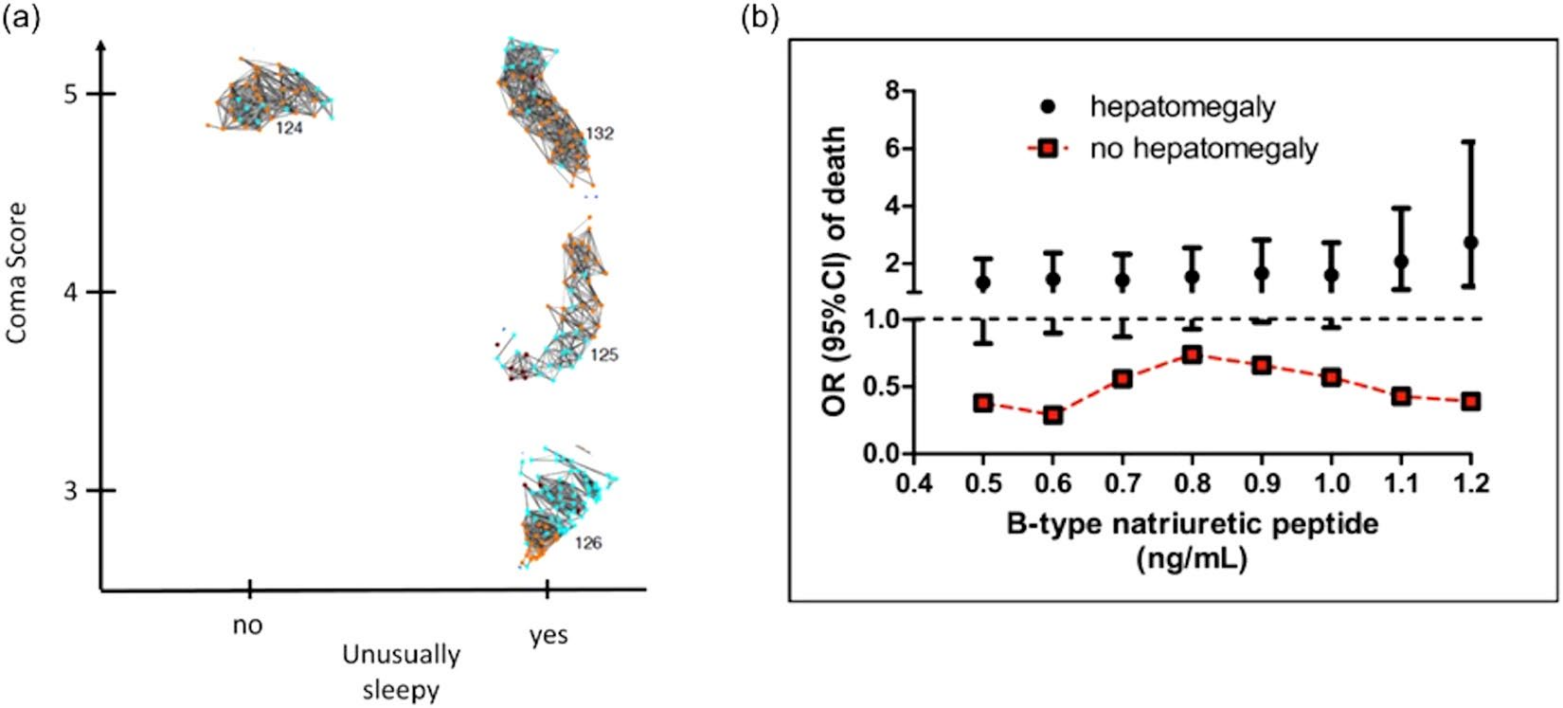
Biological validation of clusters/phenotypes associated with RD and SMA. Clinical features associated with RD and SMA clusters 124, 125, 126 and 132. Risk of death and concentration of plasma B-type natriuretic peptide (BNP) in patients with hepatomegaly (red squares) and patients without hepatomegaly (black dots). Error bars denote the 95% confidence interval of the odds of death calculated in the logistic regression analysis.

We hypothesized that increased liver size in children with severe anemia was due to impaired cardiac function (heart failure). To test this hypothesis, we measured the concentration of B-type natriuretic peptide (BNP), a biochemical marker associated with heart failure, in plasma from patients included in these four clusters (Fig. 6b). Notably, we observed that plasma BNP concentration was significantly associated with increased mortality only in those children presenting with hepatomegaly (Fig. 3). We used a fixed-effects logistic regression model to measure the association of BNP and mortality. The unadjusted model did not show any significant association (P = 0.11). However, when the analyses were stratified by presence of hepatomegaly we observed a significant increase in mortality in those children with higher BNP concentration (OR: 1.74 [95%CI 1.03–2.92], P = 0.035). These analyses were adjusted for known confounders, namely presence of respiratory distress and transfusion.

We reasoned that the phenotype revealed by the analysis of a thresholded network was not necessarily circumscribed to the cluster of interest, but rather the cluster was an indicator of a pathogenic mechanism in the population studied. We therefore compared the effect of blood transfusion in the mortality of children with and without hepatomegaly. The current WHO clinical guidelines recommend the administration of blood transfusion for children with a hemoglobin concentration up to 6 g/dL and the benefit of transfusing children with higher hemoglobin concentration is unclear. Thus, we restricted the analysis to moderately anemic children who had received blood transfusions (hemoglobin concentration from 7 g/dL to 8 g/dL) and observed that blood transfusion was associated with a 5.5 fold increase in the odds of death (OR 5.52 [95%CI 1.47–20.62] in those children with hepatomegaly but not in those without hepatomegaly (OR 0.77 [95%CI 0.17–3.37]) (sTable 3). Due to the observational nature of this study we could not establish if the survival of those children with hepatomegaly would have increased had these children not received a blood transfusion.

## Discussion

In this study, we have used a network-based clustering approach to identify a novel clinical phenotype associated with SM in Gambian children. We found that the study of clusters in this network space revealed the role of heart failure in children with severe malarial anemia and respiratory distress. These findings are clinically important and support the applicability of clustering tools to identify new clinical phenotypes in severe malaria.

To our knowledge, this is the first study that uses a network-based clustering approach to understand disease complexity in children with SM. The number of studies that have successfully used network-based tools to gain new insights into biology or clinical conditions has increased in recent years, possibly in response to the availability of high-density data derived from these models^11–13^. Here, we provide evidence that the clustering of patients in a space of eight clinical features provides a suitable scaffold to integrate new layers of biological information and to gain insight into the pathogenesis of SM. Although PCA was used as the initial step for feature selection, the underlying rationale for this approach was to identify phenotypes that were clinically important and thus only PCA-selected variables that were associated with mortality were further used to determine the variables specifying our network.

The diversity of clinical presentations of SM poses many challenges for adequate condition management. We have observed that the clusters of children with lower homogeneity (larger distances in the defined feature space) were associated with the presence of multiple SM syndromes and increased mortality rates. The current definition of SM is based on a number of clinical features associated with poor outcome but does not necessarily reflect a unique mechanism of disease^10^. Indeed, this definition captures a widely heterogeneous population of patients at high risk of dying with one or more SM syndromes, namely coma, respiratory distress and severe anemia^4,14,15^. Expectedly, the heterogeneity of patients presenting with a single SM syndrome was lower than that of patients presenting with multiple SM syndromes. However, patient mortality was significantly higher in areas with larger inter-patient distances suggesting clinical phenotypes with higher heterogeneity. Intuitively, the diagnosis and clinical management of patients who present with clinical features that depart from the ‘average’ case could be more challenging than that of the ‘standard’ patient. However, large-scale prospective clinical studies would be required to establish a causal link between cluster heterogeneity and mortality in SM.

In children with SM, respiratory distress is a major risk factor of death generally associated with metabolic complications which result directly or indirectly from insufficient oxygen tissue delivery^10^. However, the pathogenesis of RD is not completely understood^16,17^. Unexpectedly, the distribution of patients in the thresholded network revealed four clusters of children with RD and SMA: it had been expected they would lie in the clusters associated with their clinical labels. We found that hepatomegaly was the clinical feature that accounted for most of the variability between the clusters. The clinical relevance of hepatomegaly was initially unclear. Hepatomegaly was not one of the eight features selected to derive the original network and it is a non-specific clinical sign associated with a large number of conditions^18,19^. We reasoned that impaired cardiac function was a plausible mechanistic explanation for the pathogenesis of hepatomegaly.

A plasma proteomic study was conducted as biological validation of the network partition. In particular, we investigated if there was a biological correspondence of the cluster segregation observed in patients with SMA and RD (124, 125, 126 and 132). Firstly, we hypothesized that if the network partition was a random result or a ‘mathematical artefact’, the analysis of plasma samples from patients in these clusters would yield identical signatures. Secondly, we reasoned that if the proteomic signatures were different for each cluster, the proteins identified could provide an insight into the mechanism of disease associated with these clusters. Indeed, the plasma proteomic analysis of patients in these clusters supported the biological identity of these groups but was not conclusive about the role of heart failure (since molecular markers associated with impaired cardiac function are found in plasma at concentrations below the resolution achieved by standard mass spectrometry techniques^20^). We thus measured B-type natriuretic peptide (BNP) in plasma samples from patients in these clusters. BNP is a 32-amino-acid peptide synthesized primarily in the ventricles in response to ventricular wall stress and left ventricular filling pressures^21,22^. Although the concentration of plasma BNP was high in the four clusters, this molecule was associated with increased mortality only in those children with SM who were admitted with hepatomegaly. This finding supports the notion that hepatomegaly in these patients was a specific indicator of impaired heart function.

This study has limitations. Firstly, the feature space of the patient distribution was determined by few variables known to impact the clinical outcome of SM and probably missed the potential impact of other non-prognostic variables or even variables that were not collected in the case report form. Secondly, we have only used sparse PCA as a feature selection tool, which has advantages but also some important limitations. Thirdly, we have not analysed every single cluster or “clinical phenotype” in the SM network. Instead, we have selected four clusters based on the observation that these clusters corresponded to a specific “clinical phenotype” (as defined by the WHO) but were segregated. With these limitations in mind, we reasoned that the correspondence of these clusters with specific clinical features was biologically meaningful and thus, decided to test the hypothesis that heart failure could be an important feature of SM.

A limitation of plasma proteomic studies, including ours, is the broad dynamic range of protein concentrations which range from mg/ml to pg/mL (10 orders of magnitude). Standard LC-MS/MS can only identify proteins at concentrations above high ng/mL even after extensive fractionation. It is therefore possible, that a more biologically meaningful signature could have been derived with longer chromatographic gradients or further orthogonal fractionation.

The existence of heart failure in SM is controversial and critical for patient management. A number of pathogenic mechanisms commonly observed in children with SM such as hypoxia, inflammation and metabolic acidosis alone or in combination may be sufficient to impair cardiac function^10^. Indeed, evidence of myocardial dysfunction has been reported in African children with SM and adults with imported malaria^23,24^. Similarly, increased pulmonary vascular resistance which could cause right-side heart failure has been reported in patients with SM^25^. Notably, in the population studied hepatomegaly was correlated with the degree of anemia. The most severe forms of anemia were associated with lower haptoglobin concentrations suggesting ongoing erythrocyte destruction and release of free hemoglobin. These findings are compatible with previous observations suggesting that free-hemoglobin increases vascular resistance by reducing nitric oxide availability^26–28^.

Our results indicate that the role of heart failure should be reconsidered as a pathogenic mechanism in SM. In light of recent and conclusive observations suggesting that aggressive fluid management increases mortality in children with SM, we believe our findings are clinically relevant^29,30^. The clinical impact of these findings should be evaluated in prospective studies. Our data support the notion that a systems analysis of clinical features may identify new phenotypes and contribute to our understanding of disease heterogeneity. Failure to capture disease heterogeneity may underestimate the benefit of a potentially useful intervention in clinical studies. We anticipate that methods that contribute to understand disease complexity could also be valuable tools for fine-tuned patient selection in randomized controlled trials.

## Methods

### Study population

The study population consisted of 2,915 children aged 4 months to 15 years and diagnosed with severe malaria according to the WHO definition. Children were admitted to the Royal Victoria Teaching Hospital (RVTH) from January 1997 to December 2009^4,5^. The study was originally designed to study genetic variants associated with severe malaria^5^. The initial set of variables used for feature selection included those present in the case report form. The list of the variables included is described in Supplementary Table 1.

### Clinical definitions

Children aged 4 months to 15 years were eligible for enrolment if they had a blood smear positive for asexual P. falciparum parasites and met one or more WHO criteria for SM^10^: Coma (assessed by the Blantyre Coma Score [BCS]^7^), severe anemia (hemoglobin [Hb] <50 g/L or packed cell volume [PCV] <15), respiratory distress (costal indrawing, use of accessory muscles, nasal flaring, deep breathing), hypoglycemia (<2.2 mM), decompensated shock (systolic blood pressure less than 70 mmHg), repeated convulsions (>3 during a 24 hour-period), acidosis (plasma bicarbonate <15 mmol/L) and hyperlactatemia (plasma lactate >5 mmol/L). CM was defined as a BCS of 2 or less with any P.falciparum parasite density. Hepatomegaly was defined as >2 cm of palpable liver below the right costal margin. Patients were enrolled in the study if informed consent was given by the parent or guardian. The study protocol was approved by the Joint Gambia Government/MRC Ethical Committee (protocol numbers 630 and 670).

### Laboratory measurements

Hemoglobin was measured with a hematology analyzer (Coulter ® MD II, Coulter Corporation, USA) and parasite density was counted on Giemsa-stained thick and thin films. Plasma samples were collected and stored following Good Clinical and Laboratory Practice protocols (GCLP) at the MRC Laboratories (Gambia) and only thawed once to generate aliquots. Plasma concentration of B-type natriuretic peptide (BNP) was measured using commercially available immunoassay (Phoenix Europe, Germany) following manufacturer’s instructions.

### Data management and statistical analyses

The data were collected on standardized forms, double entered into a database and verified against the original. The original dataset did not contain a large proportion of missing values (median of missing values of 3.7% and average of 15.8% for different variables). We chose to impute the missing values in order to preserve the largest possible number of variables and patients. Given that the percentage of missing values was small, and making the assumption that data were missing at random, we used the simple and widely used column mean imputation to impute the missing values. Different imputation techniques were assessed, including mean and KNN imputation, and since the results were similar (Rand Index of partitionings above 0.80), we were confident that the choice of missing value imputation method did not impact the results. Univariate and multivariate logistic regression models were fitted for clinical variables to identify clinical features associated with clinical outcome using Stata (v11). The analytical tools described in the following sections were implemented in Matlab (R2010a) and some visualisations were performed using the statistical environment R (3.5.0).

### Feature selection and thresholding

Sparse PCA was used to select clinical features to define the matrix of Euclidean pairwise distances between all the data points (patients)^6^. All variables were normalized prior to analysis. Pairs of data points with a Euclidean distance below a given threshold were connected to form an unweighted network (see connectivity of the network in sFig. 3). The distance threshold was chosen to be the first local maximum of the average clustering coefficient as the threshold was increased from zero to the maximum pairwise distance (sFig. 2). This analysis attempts to recover a natural scale at which the data points form relatively tight small clusters of patients, where clusters are defined as the distinct connected components recovered after thresholding. Pairwise distances and thresholded networks have been successfully used previously to address comparable complex networks, ranging from social sciences to genetics^31–33^.

A similarity matrix containing all the distances between any pair of points (patients) in the dataset was constructed in the eight-dimensional space determined by the 8 clinical features selected. In this matrix, smaller entries/distances indicated a greater similarity between patients in their clinical presentation. To maximize similarities within clusters and differences between clusters of patients, an appropriate distance threshold was defined as a fraction of the maximum pairwise distance between patients. We sought to find a distance threshold that maximized the average clustering coefficient but generated a partition with components of sufficient size to derive meaningful statistical analysis in relation to clinical outcome (Online methods and sFigs 2, 3). We took a distinctive but simple approach and treated each different connected component in the thresholded network as a cluster (we plotted the network using a force (spring)-based algorithm network visualization method).

### Calculation of Gaussian density function

A Gaussian kernel density function was used to measure closeness of patients in the clinical feature space^34^. The calculation of individual density for each patient was used to measure proximity of patients in the unthresholded clinical feature space. This calculation was thus independent of cluster allocation in the thresholded network. The density function was defined as follows:

$${D}_{x}=C\sum _{y}{e}^{\frac{-d{(x,y)}^{2}}{\sigma }}$$

Dx is the density associated with patient x, calculated as the sum over all contributions from Gaussian kernels centered at every other patient y where d(x, y) is the Euclidean distance and where C corresponds to the Gaussian kernels’s normalization constant. The variance selected (σ = 0.2) included all inter-patient distances.

### Proteomic analysis

Plasma samples from 140 Gambian children aged 2 to 59 months were used for proteomic studies. Samples were obtained from patients included in clusters 124, 125, 126 and 132. Individual samples (5 µl of plasma) were pooled into 3 different groups (~55 µl of plasma per batch) in each cluster category: 35 individual samples were randomly divided into three groups of 11, 12 and 12 samples (see Fig. 2c). Pooled plasma samples were depleted of the top 14 highly-abundant plasma proteins with a multiple affinity removal (MARS) column (Agilent, UK) using high-performance liquid-chromatography (HPLC) 1200 series (Agilent, UK). Proteins from depleted plasma were precipitated with trichloroacetic acid and quantified using a colorimetric assay (BCA Protein assay, Thermo Scientific, US) and further separated by size using SDS-PAGE and bands were cut and digested with trypsin. Peptide digests were purified using Sep-Pak C18 columns (Waters, Milford, MA). The nano-LC system (final rate 0.3 μl/min) was coupled to *LTQ-*Orbitrap Velos (Thermo) and searched against the human proteome with a false-discovery rate of 1% calculated from target-decoy hits and relative (label-free) quantification was based on normalized spectral index quantitation (SINQ)^35^.

## Electronic supplementary material

Supplementary information
