# Supplementary material for: Identification of a Novel Clinical Phenotype of Severe Malaria using a Network-Based Clustering Approach
Source: Sci Rep. 2018 Aug 27;8:12849. doi: 10.1038/s41598-018-31320-w (PMC6110866; doi:10.1038/s41598-018-31320-w)
Supplement: Supplementary file 1 — Supplementary information [file 41598_2018_31320_MOESM1_ESM.docx]

**Supplementary materials**

**Identification of a Novel Clinical Phenotype of Severe Malaria using a Network-Based Clustering Approach**

Ornella Cominetti, David Smith, Fred Hoffman, Muminatou Jallow, Marie L Thézénas, Honglei Huang, Dominic Kwiatkowski, Philip K Maini and Climent Casals-Pascual.

**Supplementary Table 1|** List of clinical variables included in the study

| **Number** | **Variables** | **Unit** | **Value range** | **sPCA** | **MV%** |
| --- | --- | --- | --- | --- | --- |
| 1 | **Blantyre Coma score** | score | 0-5 | x | 0.2% |
| 2 | Coma | Yes/no | 1/0 | x | 3.3% |
| 3 | **Convulsions during admission** | Yes/no | 1/0 | x | 65.4% |
| 4 | Cough | Yes/no | 1/0 | x | 2.6% |
| 5 | **Deep breathing** | Yes/no | 1/0 | x | 64.8% |
| 6 | **Hb concentration** | g/dL | 1-15.24 | x | 2.1% |
| 7 | **Intercostal recession** | Yes/no | 1/0 | x | 70.5% |
| 8 | Irritability | Yes/no | 1/0 | x | 4.2% |
| 9 | Lethargy | Yes/no | 1/0 | x | 4.0% |
| 10 | **Posture/tonic seizures** | Yes/no | 1/0 | x | 77.0% |
| 11 | Posturing | Yes/no | 1/0 | x | 22.3% |
| 12 | **Unusually sleepy** | Yes/no | 1/0 | x | 1.7% |
| 13 | **Use of accessory respiratory muscles** | Yes/no | 1/0 | x | 2.2% |
| 14 | Age | months | 4-180 |  | 0.4% |
| 15 | Coma duration | hours | 0-96 |  | 49.7% |
| 16 | Death | Yes/no | 1/0 |  | 0.4% |
| 17 | Dehydration | Yes/no | 1/0 |  | 21.2% |
| 18 | Diarrhea | Yes/no | 1/0 |  | 2.3% |
| 19 | ^§^Ethnic group |  | M, W, F, J, S, O |  | 14.0% |
| 20 | Father alive | Yes/no | 1/0 |  | 0.8% |
| 21 | Fever duration | days | 1/0 |  | 8.7% |
| 22 | Grunting | Yes/no | 1/0 |  | 2.5% |
| 23 | Heart rate | bpm | 12-190 |  | 0.3% |
| 24 | Hypoglycemia | Yes/no | 1/0 |  | 29.9% |
| 25 | Irregular breathing | Yes/no | 1/0 |  | 2.6% |
| 26 | Jaundice | Yes/no | 1/0 |  | 2.2% |
| 27 | Liver size | cm | 0-6 |  | 1.2% |
| 28 | Mother alive | Yes/no | 1/0 |  | 0.5% |
| 29 | Neurological sequelae | Yes/no | 1/0 |  | 21.7% |
| 30 | Number of seizures | number | 0-20 |  | 40.5% |
| 31 | *P. falciparum* /µL | number/µL | 50-500,000 |  | 2.1% |
| 32 | Pallor | Yes/no | 1/0 |  | 32.1% |
| 33 | Prostration | Yes/no | 1/0 |  | 23.3% |
| 34 | Pulmonary edema | Yes/no | 1/0 |  | 5.9% |
| 35 | Refuse to feed | Yes/no | 1/0 |  | 2.3% |
| 36 | Renal failure | Yes/no | 1/0 |  | 5.9% |
| 37 | Respiratory rate | bpm | 18-98 |  | 0.5% |
| 38 | Saturation of oxygen | % | 20-100 |  | 66.1% |
| 39 | Seizures (reported) | Yes/no | 1/0 |  | 5.5% |
| 40 | Sex | male/female | 1/0 |  | 0.0% |
| 41 | Spleen size | cm | 0-5 |  | 1.2% |
| 42 | Temperature | °C | 35-42.2 |  | 1.1% |
| 43 | Transfusion | Yes/no | 1/0 |  | 4.5% |
| 44 | Venous pulse | Yes/no | 40-300 |  | 26.1% |
| 45 | Vomit | Yes/no | 1/0 |  | 2.1% |
| 46 | Weight-for-age Z-score | Z-score | -4.22 to +7.8 |  | 29.4% |

§ Ethnic group (M=Mandinka, W=Wolof, F=Fula, J=Jola, S=Serahuli, O=Other)

**Supplementary Table 2**| Selection of clinical features to calculate patient pairwise distances (adjacency matrix). Data show Pearson’s correlation coefficient corresponding to 13 clinical features and outcome (death). * P<0.001 ‡ Coma is defined as Blantyre coma score (BCS) <3. BCS but not coma was selected to generate the adjacency matrix.


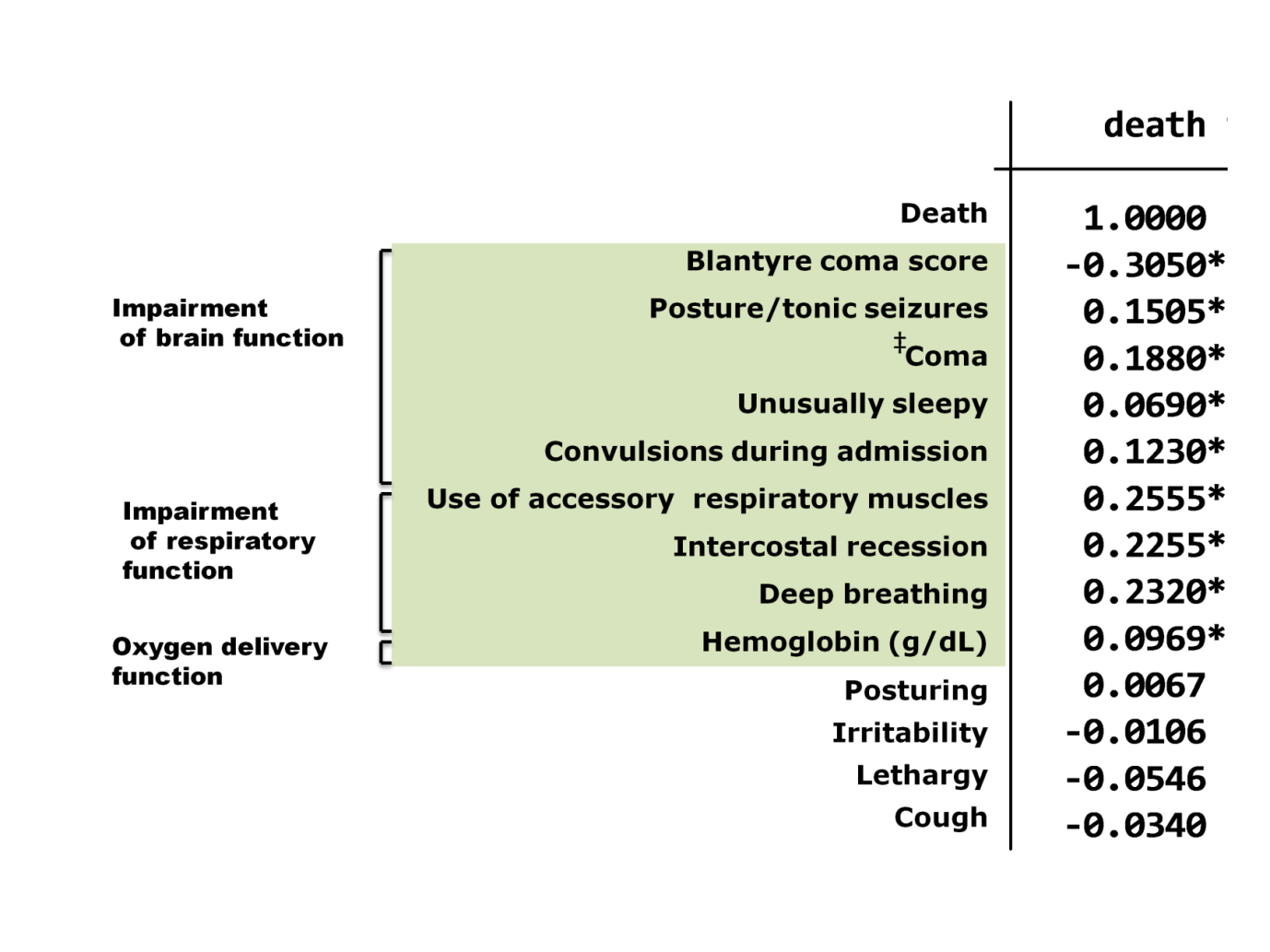


**Supplementary Table 3.** Logistic regression model to evaluate the effect of transfusion of mortality (dependent variable: death) stratified by the presence of hepatomegaly on admission and adjusted for respiratory distress (rd). Patients with Blantyre coma scores <3 and moderate or severe anemia are excluded from the analyses. Hepato: hepatomegaly; tfus: transfusion; rd: respiratory distress; std. Err: standard error; 95%C Conf. Interval: 95% Confidence interval.

**Supplementary Table 4. Clinical and laboratory features of the clusters analysed.**

|  |  | Cluster 124 | Cluster 132 | Cluster 125 | Cluster 126 |
| --- | --- | --- | --- | --- | --- |
| Observations | N | 38 | 48 | 48 | 83 |
| Mortality | (%) | 3.03 | 11.3 | 16.2 | 20.2 |
| Age (months) | Median  (IQR) | 20.5  (11-31) | 20.5  (12-32) | 26.5  (13-46) | 38  (24-55) |
| Hemoglobin (g/dL) | Median  (IQR) | 4.3  (3.8-5.0) | 4.4  (3.5-5.0) | 5.0  (3.5-5.9) | 6.8  (5.0-8.3) |
| Parasite density (number/µL) | Geom. Mean  (95%CI) | 37,830  (20,040-71,412) | 26,026  (13,557-49,963) | 54,960  (33,116-91,213) | 49,674  (32,894-75,014) |
| Transfusion | (%) | 100 | 100 | 75 | 52 |
| Respiratory distress | (%) | 100 | 97.9 | 89.5 | 96.3 |
| Hepatomegaly | (%) | 54 | 73 | 58 | 40 |
| Cough | (%) | 54 | 80 | 46 | 28 |
| BNP (ng/mL) | Median  (IQR) | 1.2  (1.0-1.5) | 1.4  (1.0-1.94) | 1.2  (0.9-1.7) | 1.0  (1.0-1.9) |

BNP: B-type natriuretic peptide

**Supplementary Figure 1.** Feature selection. The initial set of clinical features was selected based on their ability to predict data variability using sparse (a) and standard PCA analysis (b) and their ability to predict death in a multiple logistic regression analysis with an appropriate significance level (*P<0.01) and a low collinearity measure by their low variance inflation factor (dotted line) (c). PCA: principal component analysis; AUROC MLR: area under the curve of the multiple logistic regression analysis using death as a dependent variable; BCS: Blantyre coma score.

(a)

(b)


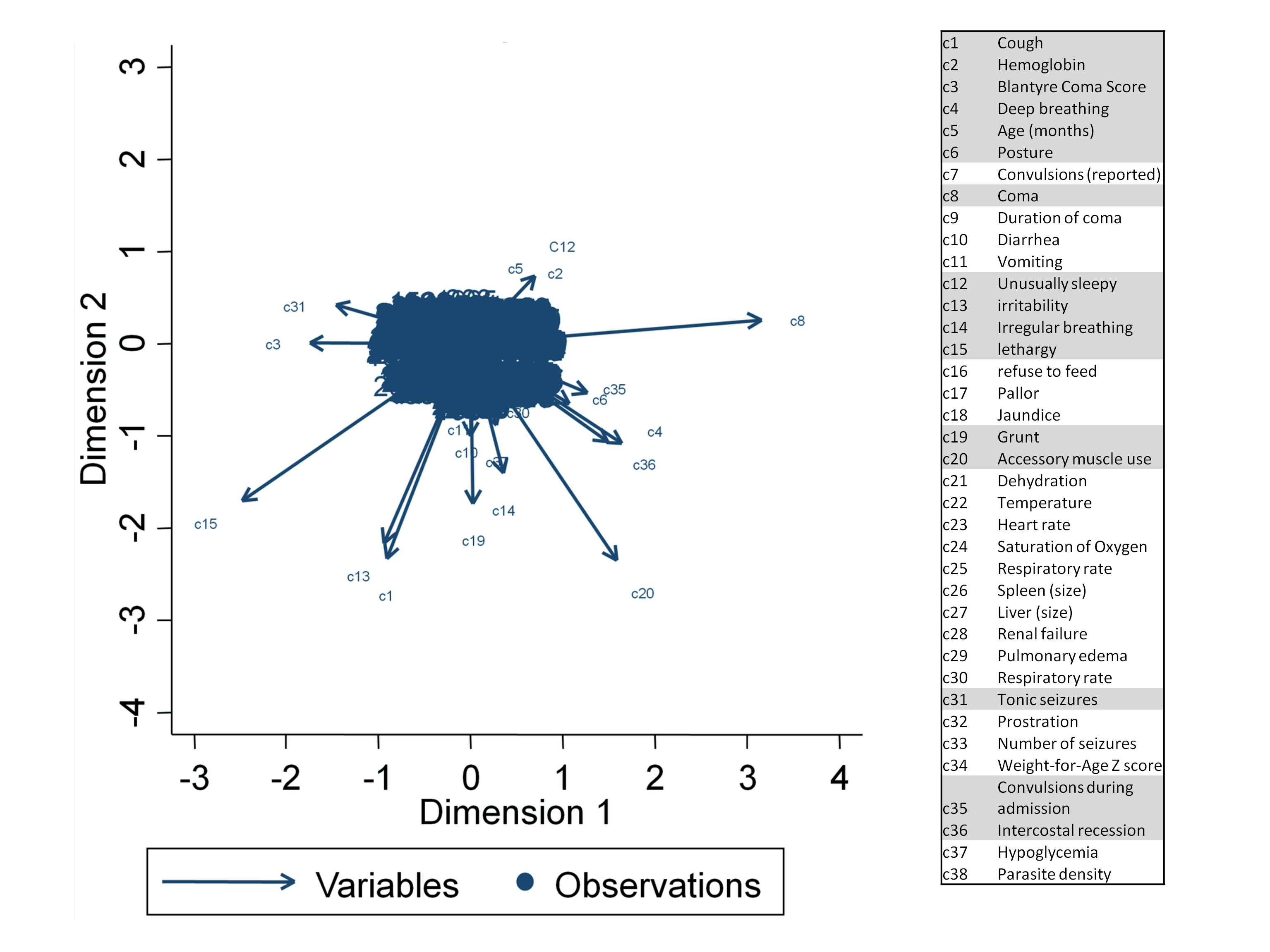


(c )


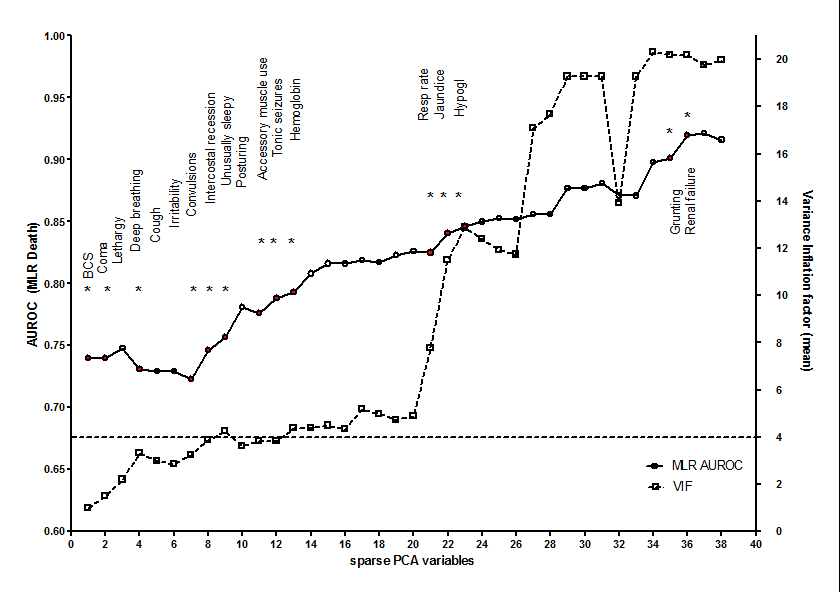


**Supplementary Figure 2.** Comparison of the clustering coefficient and threshold of the network based on 8 selected features with that of 100 randomly generated networks. Average clustering coefficient was plotted against normalized pairwise distance thresholds. Clustering coefficients were calculated for the eight-feature derived network (blue) and for randomised networks with a degree distribution identical to the original network for each computed threshold (green). In the original network derived from the 8 features selected, distance threshold values above 0.3 resulted in a large connected component that included 80% or more of the population studied in a single cluster (diamonds). For threshold values below 0.3, the highest average clustering coefficient obtained was 0.05.


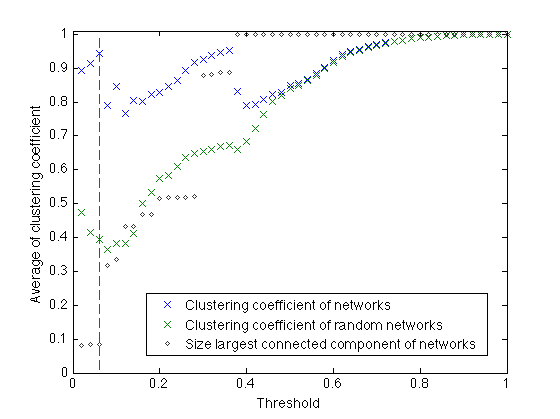


**Supplementary Figure 3.** Adjacency matrices derived for 2,915 patients using the clinical features selected by sparse PCA. Data show adjacency matrices for different distance thresholds: 0.8%, 2.5%, 5% and 10% of the maximum pairwise distance.


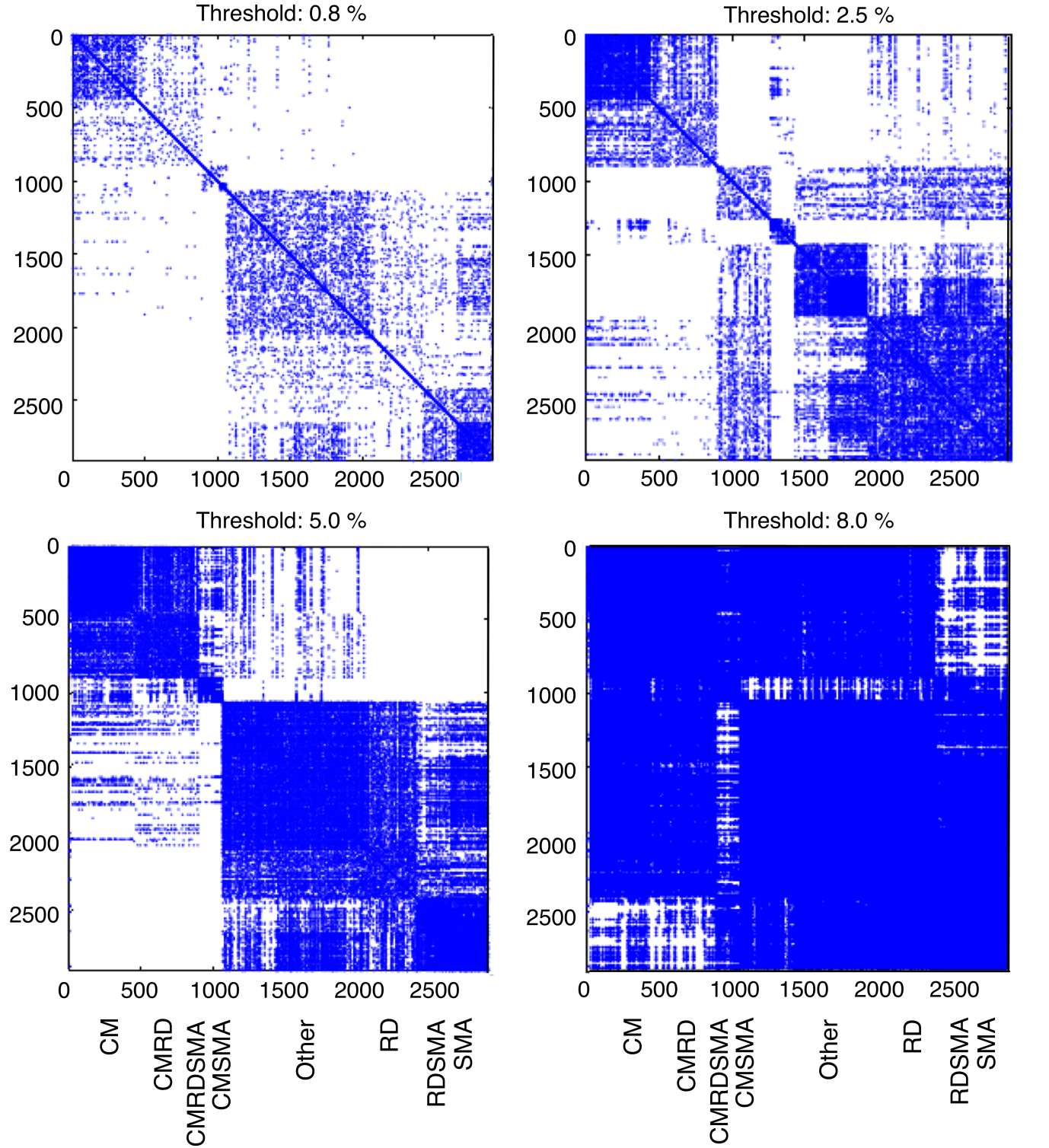


**Supplementary Figure 4.** **Connected components and clinical outcome**. To investigate if knowledge of the cluster in which a patient lies is informative of patient clinical outcome, the mortality of each cluster (>20 nodes/patients) of the eight-feature derived network was calculated (triangles) and compared with that of 1,000 randomly-generated random networks (in grey). Random networks were generated by shuffling the nodes but preserving the topology and vertex distribution of the original thresholded network ([1](#_ENREF_1)). Blue squares indicate randomly generated clusters, the average and the 95% confidence interval (blue error bars) of the cluster-associated mortality. The average mortality in the study population was 13%.


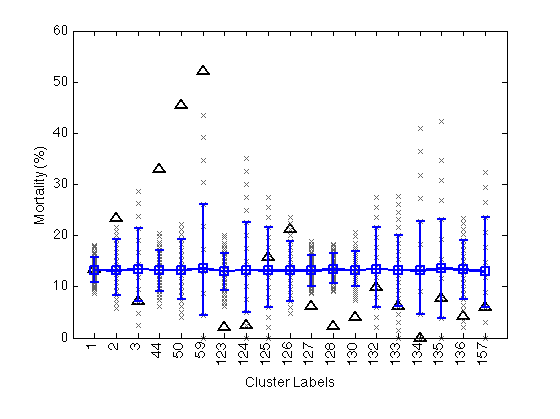


**Supplementary Figure 5.** Validation of respiratory distress and severe anemia clusters. Using hierarchical clustering with average linkage, K = 150 (shown in red dotted vertical line in (a)), a notional network was built by linking all co-clustered pairs of points (b) (left). From such a network, we identified the respiratory distress and severe anaemia clusters from the original network shown in Figure 2a (right). We used the Rand Index (RI)([2](#_ENREF_2)) as a measure of the similarity between the two clusters (a RI of 1 indicates that two partitions agree perfectly). A RI of 0.98 was obtained indicating excellent agreement between both networks. The figure in (a) shows that the region around K=150 has a very high average silhouette width, near the maximum obtained with this clustering technique.

(a)

(b)


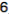

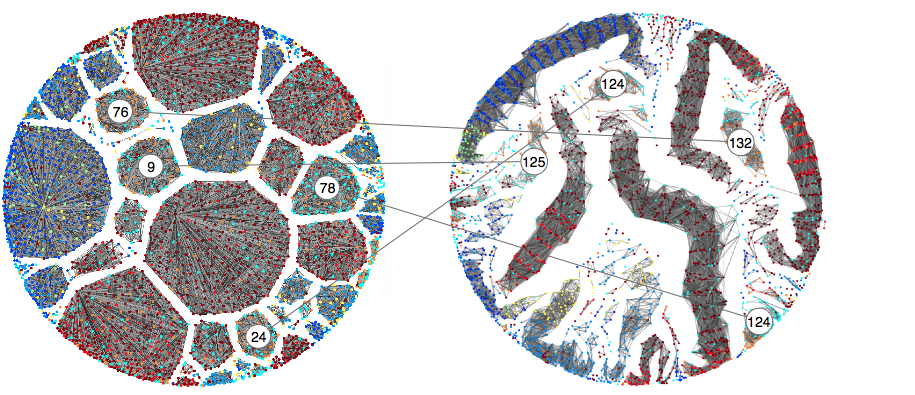


**Supplementary Figure 6. Density values in patients according to clinical syndrome of SM**. (a) Distribution dotplot shows density values of children with different SM syndromes that survived (green) and children who died (red) and median (IQR) values for each SM syndrome (b). Distribution of all Euclidean distances for the population studied. Threshold indicates the cut-off value used to plot the thresholded network (c).


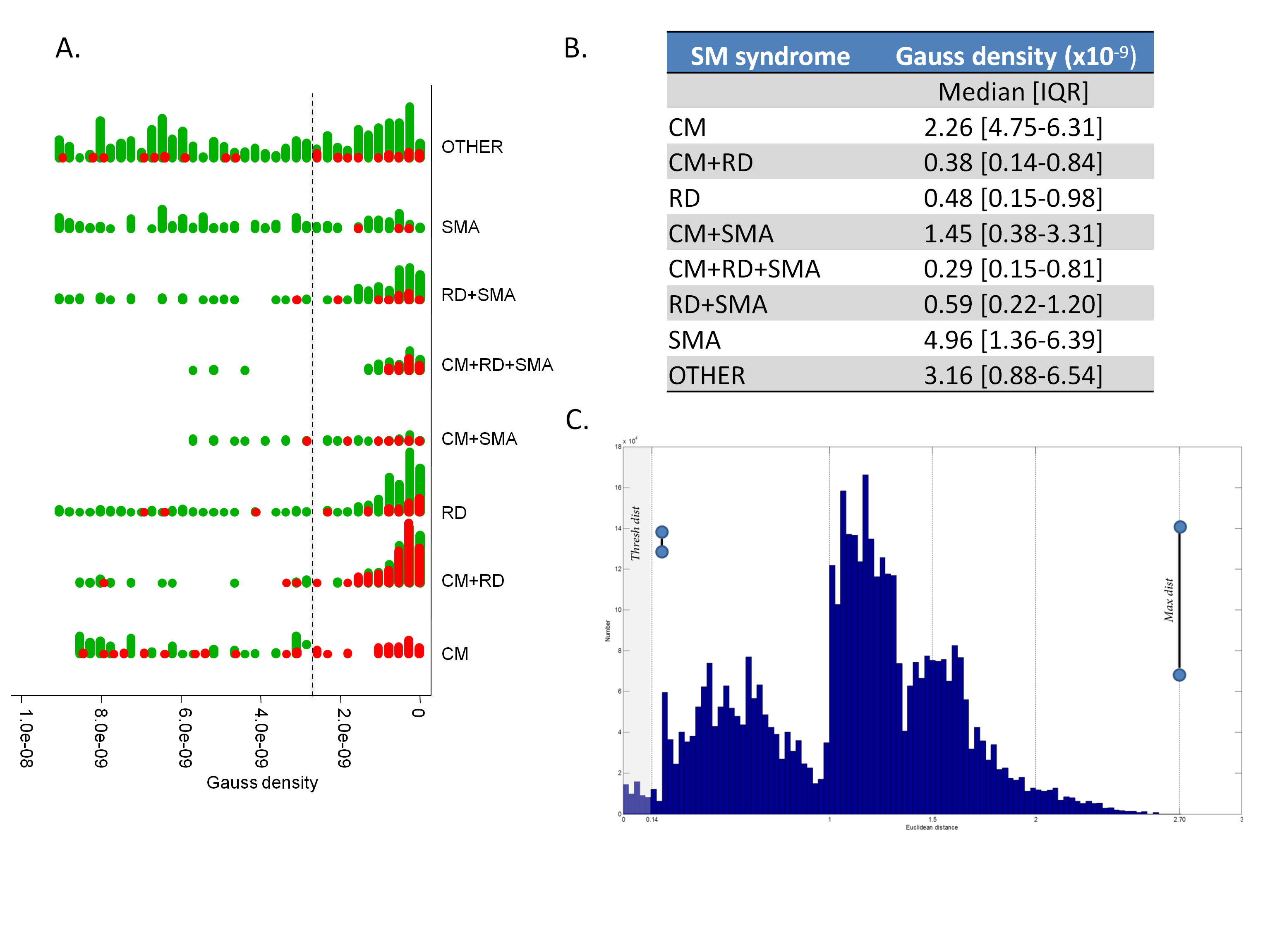


**Supplementary Figure 7. Biological validation of clusters/phenotypes associated with RD and SMA.**  (a) Characterization of the plasma proteome of children from SM clusters with RD and SMA. The proteomic analysis of samples from patients in clusters 124, 125, 126 and 132 was carried out using liquid-chromatography tandem mass spectrometry (LC-MS/MS). Plasma samples were depleted of the 14 most abundant proteins and further fractionated using SDS-PAGE to increase proteome dynamic range. Hierarchical clustering (average linkage) shows normalized protein abundance based on label-free quantification using normalized spectral index (SINQ)([3](#_ENREF_3)).

**
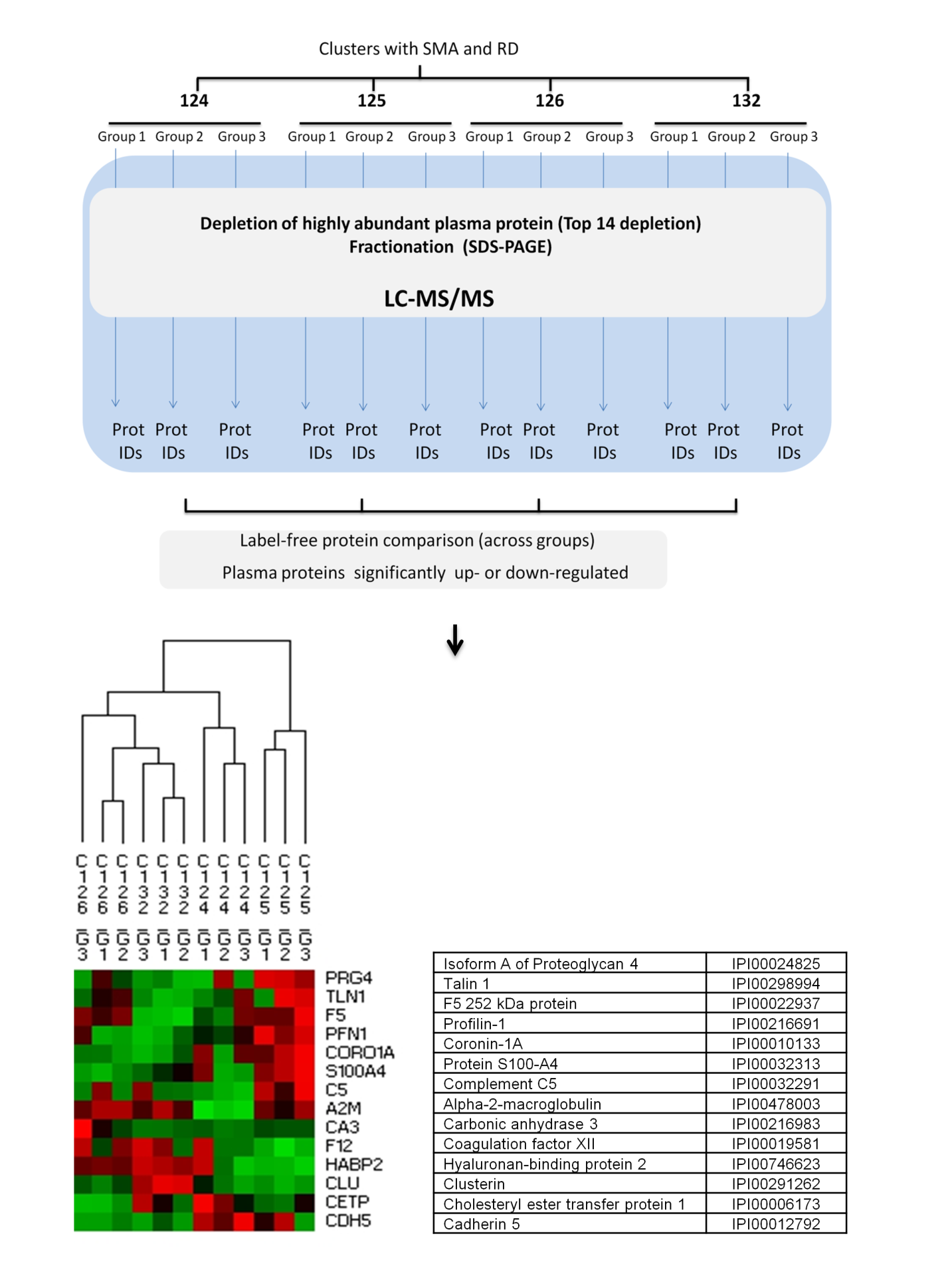
**

**References**

1. Li J*, et al.* (2009) Network-assisted protein identification and data interpretation in shotgun proteomics. *Mol Syst Biol* 5:303.

2. Rand WM (1971) Objective criteria for the evaluation of clustering methods. *J Amer Stat Assoc* 66(336):846-850.

3. Trudgian DC*, et al.* (2010) Comparative evaluation of label-free SINQ normalized spectral index quantitation in the central proteomics facilities pipeline. *Proteomics* 11(14):2790-2797.
